# Supplementary material for: Preliminary Assessment of the Protective and Antitumor Effects of Several Phytoene-Containing Bacterial and Microalgal Extracts in Colorectal Cancer
Source: Molecules. 2024 Oct 22;29(21):5003. doi: 10.3390/molecules29215003 (PMC11547717; doi:10.3390/molecules29215003)
Supplement: Supplementary file 1 [file molecules-29-05003-s001.zip › molecules-3158810-supplementary.pdf]

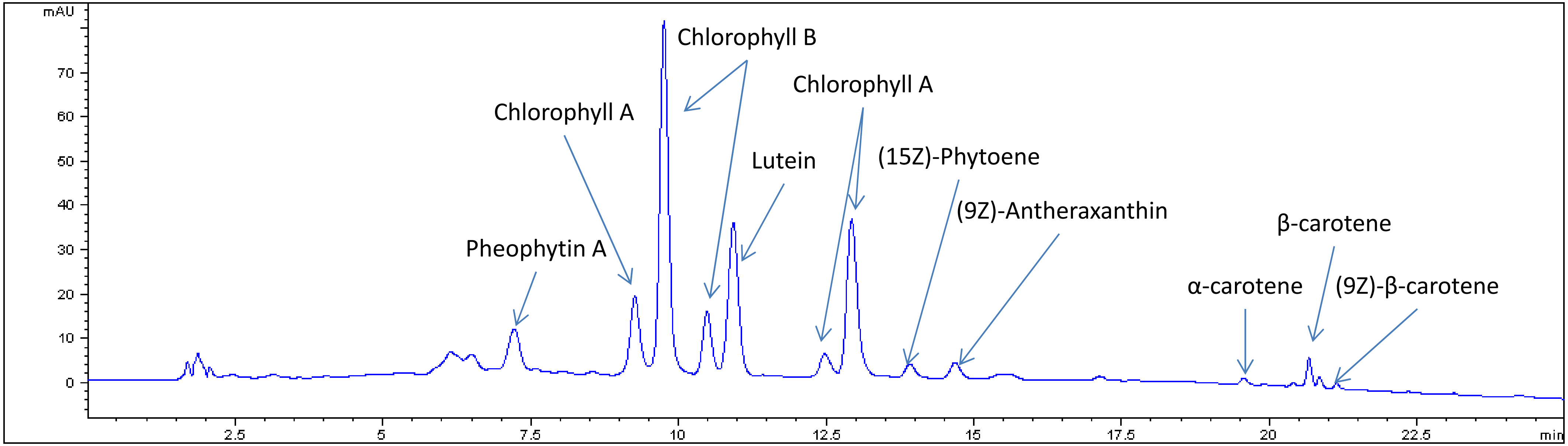

Figure S1: HPLC chromatogram of the methanolic extract obtained form the chlorophyte microalga *Chlorella sorokinina* cultured in the presence of norflurazon at a detection wavelength of 450 nm.

Chlorella sorokiniana phytoene – 285 nm

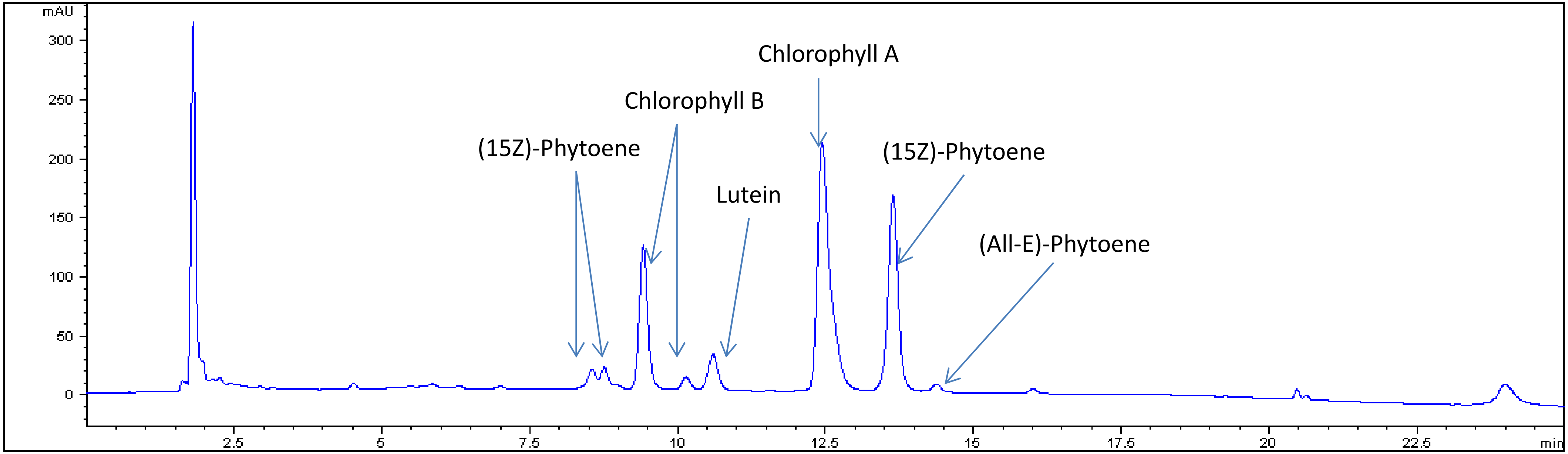

Figure S2: HPLC chromatogram of the methanolic extract obtained form the chlorophyte microalga *Chlorella sorokinina* cultured in the presence of noflurazon at a detection wavelength of 285 nm.

Sphingomonas echinodes – 285 nm

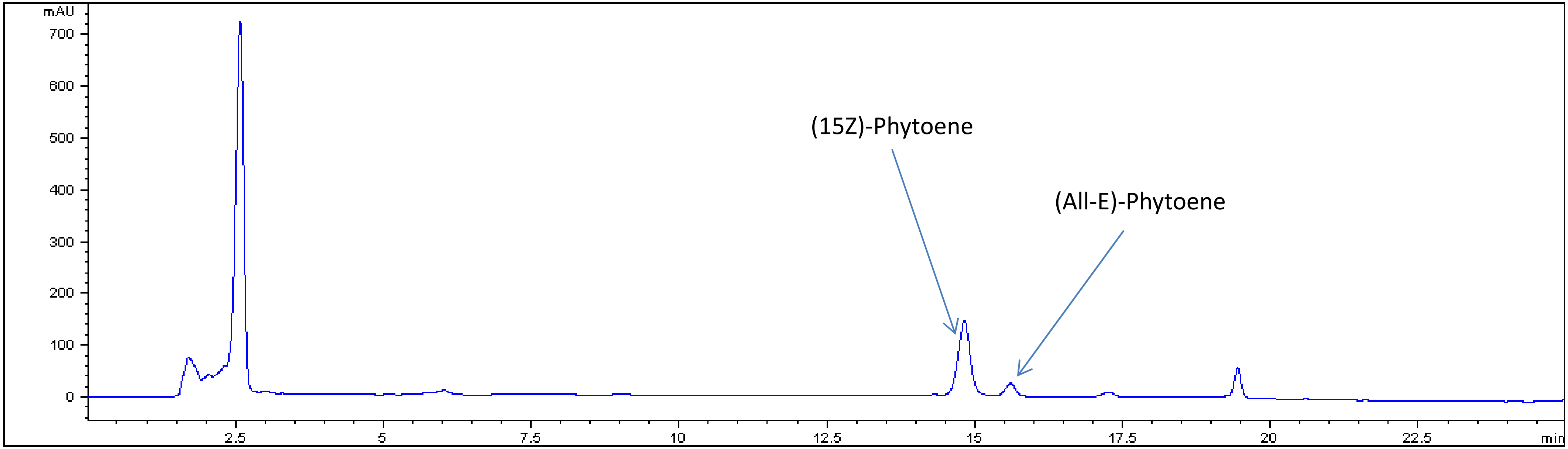

Figure S3: HPLC chromatogram of the methanolic extract obtained form the bacterium Sphingomonas echinodes at a detection wavelength of 285 nm.

Staphylococcus haemolyticus – 285 nm

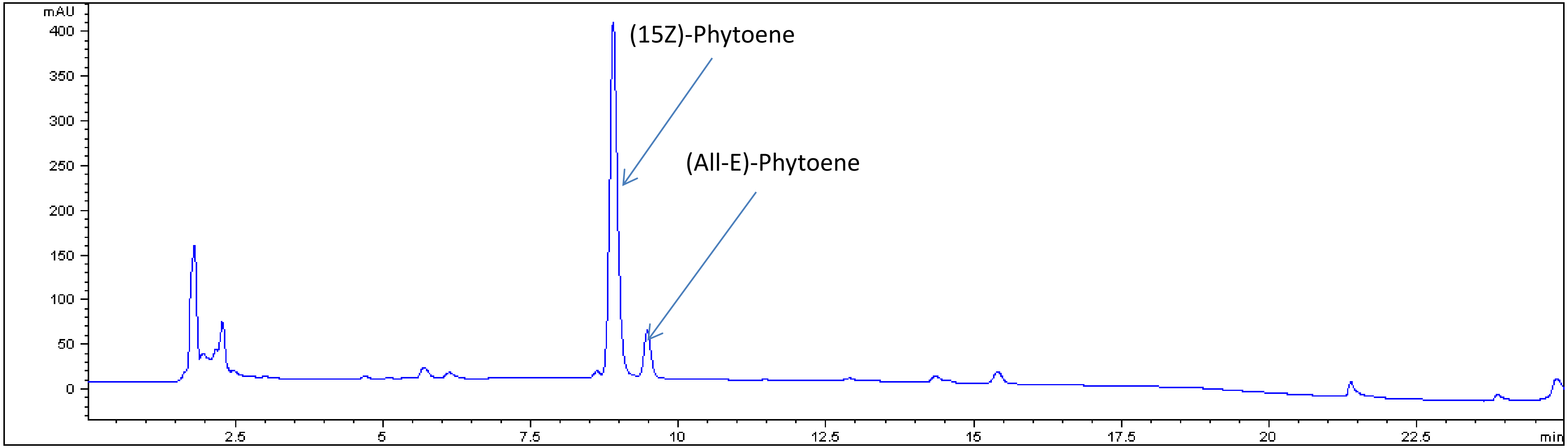

Figure S4: HPLC chromatogram of the methanolic extract obtained form the bacterium *Staphylococcus haemolyticus* at a detection wavelength of 285 nm.

Escherichia coli with pAC-PHYTipi – 285 nm

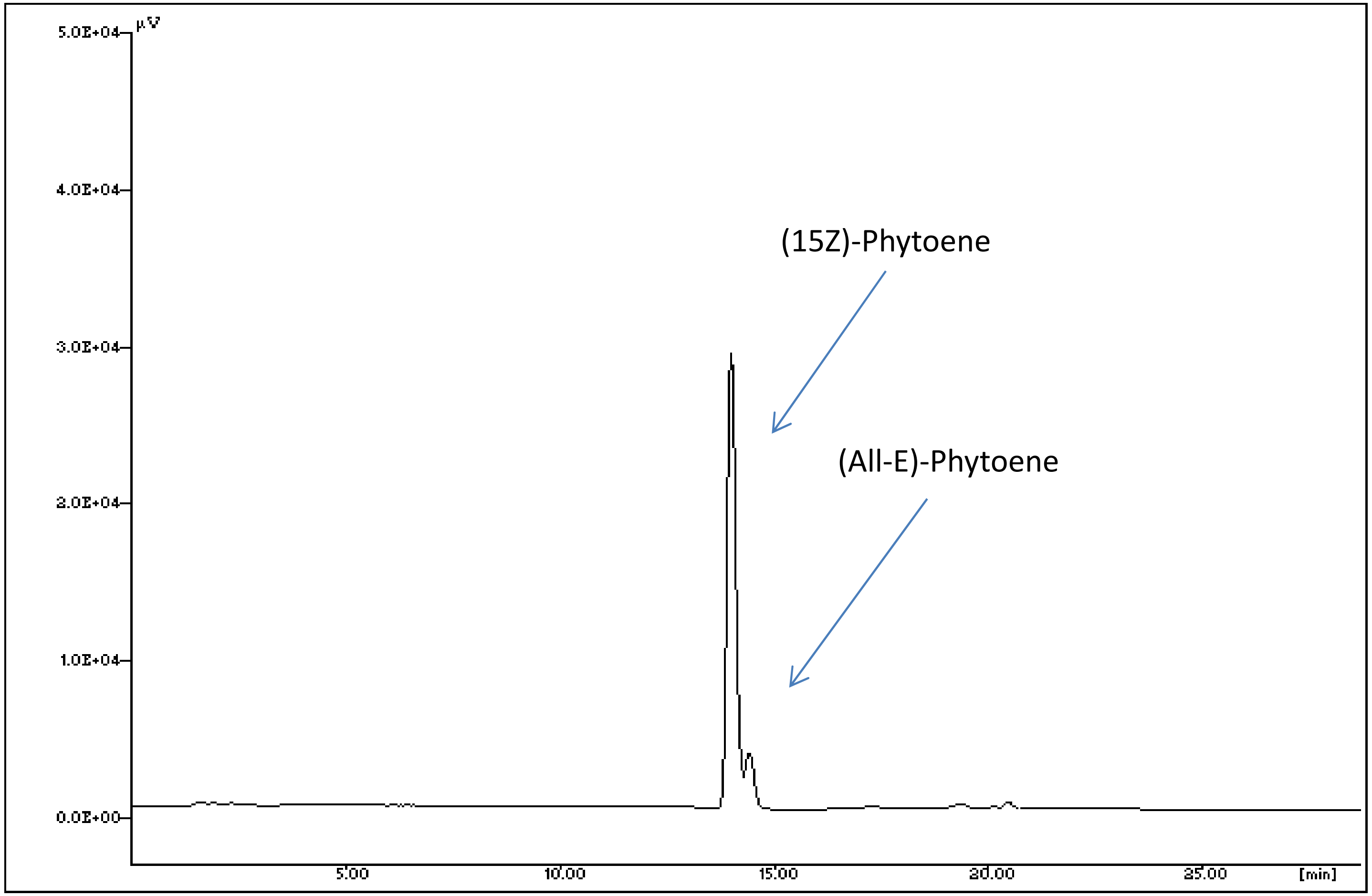

Figure S5: HPLC chromatogram of the methanolic extract obtained form the bacterium *Escherichia coli* with pAC-PHYTipi at a detection wavelength of 285 nm.
